# Imaging Biomarkers of Glioblastoma Treatment Response: A Systematic Review and Meta-Analysis of Recent Machine Learning Studies

**DOI:** 10.3389/fonc.2022.799662

**Published:** 2022-01-31

**Authors:** Thomas C. Booth, Mariusz Grzeda, Alysha Chelliah, Andrei Roman, Ayisha Al Busaidi, Carmen Dragos, Haris Shuaib, Aysha Luis, Ayesha Mirchandani, Burcu Alparslan, Nina Mansoor, Jose Lavrador, Francesco Vergani, Keyoumars Ashkan, Marc Modat, Sebastien Ourselin

**Affiliations:** ^1^School of Biomedical Engineering & Imaging Sciences, King’s College London, St. Thomas’ Hospital, London, United Kingdom; ^2^Department of Neuroradiology, King’s College Hospital National Health Service Foundation Trust, London, United Kingdom; ^3^Department of Radiology, Guy’s & St. Thomas’ National Health Service Foundation Trust, London, United Kingdom; ^4^Department of Radiology, The Oncology Institute “Prof. Dr. Ion Chiricuţă” Cluj-Napoca, Cluj-Napoca, Romania; ^5^Department of Radiology, Buckinghamshire Healthcare National Health Service Trust, Amersham, United Kingdom; ^6^Department of Medical Physics, Guy’s & St. Thomas’ National Health Service Foundation Trust, London, United Kingdom; ^7^Institute of Psychiatry, Psychology & Neuroscience, King’s College London, London, United Kingdom; ^8^Department of Radiology, Cambridge University Hospitals National Health Service Foundation Trust, Cambridge, United Kingdom; ^9^Department of Radiology, Kocaeli University, İzmit, Turkey; ^10^Department of Neurosurgery, King’s College Hospital National Health Service Foundation Trust, London, United Kingdom

**Keywords:** glioblastoma, machine learning, monitoring biomarkers, meta-analysis, artificial intelligence, treatment response, deep learning, glioma

## Abstract

**Objective:**

Monitoring biomarkers using machine learning (ML) may determine glioblastoma treatment response. We systematically reviewed quality and performance accuracy of recently published studies.

**Methods:**

Following Preferred Reporting Items for Systematic Reviews and Meta-Analysis: Diagnostic Test Accuracy, we extracted articles from MEDLINE, EMBASE and Cochrane Register between 09/2018–01/2021. Included study participants were adults with glioblastoma having undergone standard treatment (maximal resection, radiotherapy with concomitant and adjuvant temozolomide), and follow-up imaging to determine treatment response status (specifically, distinguishing progression/recurrence from progression/recurrence mimics, the target condition). Using Quality Assessment of Diagnostic Accuracy Studies Two/Checklist for Artificial Intelligence in Medical Imaging, we assessed bias risk and applicability concerns. We determined test set performance accuracy (sensitivity, specificity, precision, F1-score, balanced accuracy). We used a bivariate random-effect model to determine pooled sensitivity, specificity, area-under the receiver operator characteristic curve (ROC-AUC). Pooled measures of balanced accuracy, positive/negative likelihood ratios (PLR/NLR) and diagnostic odds ratio (DOR) were calculated. PROSPERO registered (CRD42021261965).

**Results:**

Eighteen studies were included (1335/384 patients for training/testing respectively). Small patient numbers, high bias risk, applicability concerns (particularly confounding in reference standard and patient selection) and low level of evidence, allow limited conclusions from studies. Ten studies (10/18, 56%) included in meta-analysis gave 0.769 (0.649-0.858) sensitivity [pooled (95% CI)]; 0.648 (0.749-0.532) specificity; 0.706 (0.623-0.779) balanced accuracy; 2.220 (1.560-3.140) PLR; 0.366 (0.213-0.572) NLR; 6.670 (2.800-13.500) DOR; 0.765 ROC-AUC.

**Conclusion:**

ML models using MRI features to distinguish between progression and mimics appear to demonstrate good diagnostic performance. However, study quality and design require improvement.

## 1 Introduction

**Figure 1:**
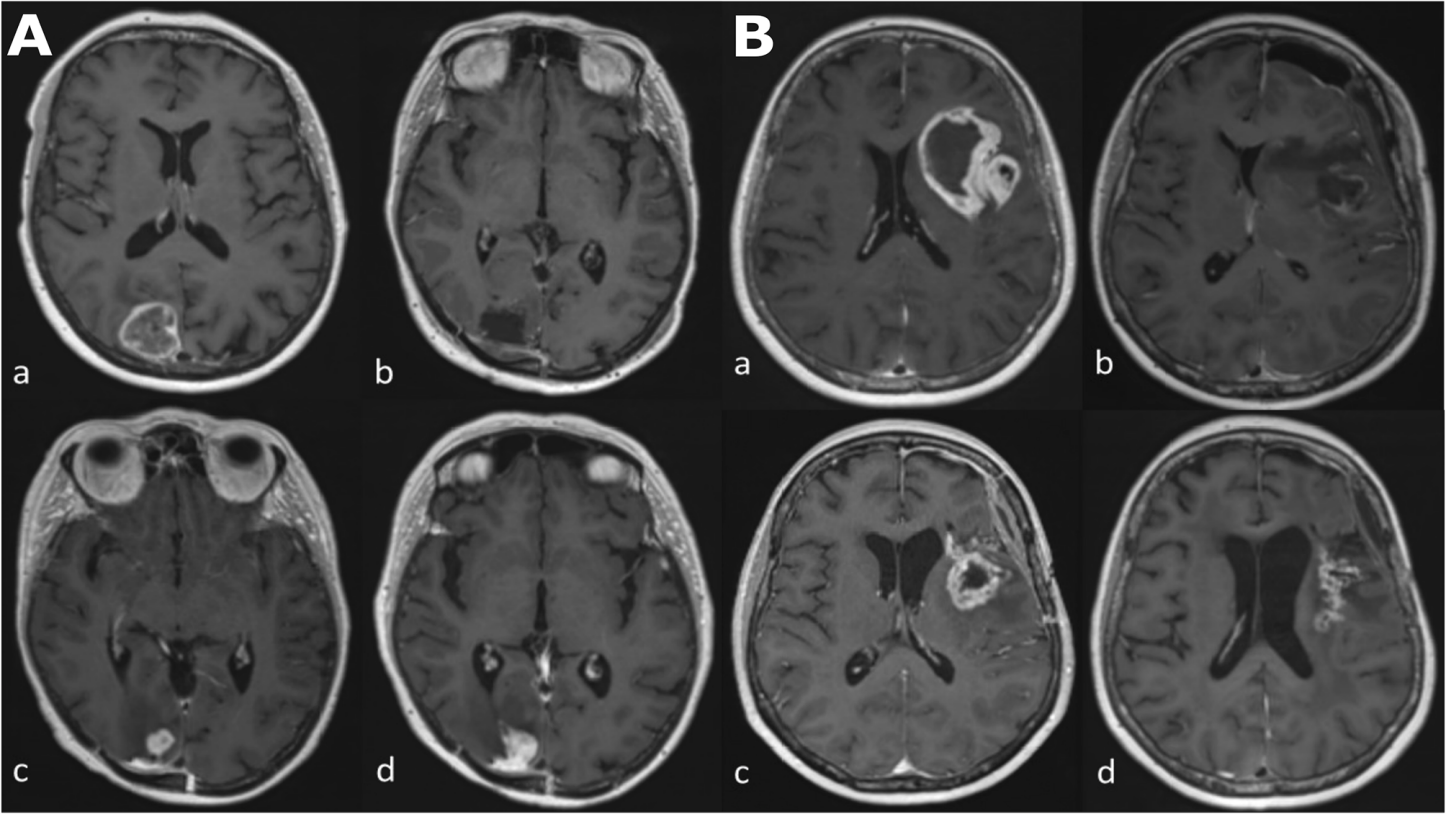
Longitudinal series of MRI images in two patients **(A, B)** with glioblastoma, IDH-wildtype. All images are axial *T*_1_-weighted after contrast administration. Images **(Aa–Ad)** demonstrate tumor progression. **(Aa)** Pre-operative MRI of a glioblastoma in the occipital lobe. **(Ab)** Post-operative MRI five days after resection; there is no contrast enhancement therefore no identifiable residual tumor. **(Ac)** The patient underwent a standard care regimen of radiotherapy and temozolomide. A new enhancing lesion at the inferior margin of the post-operative cavity was identified on MRI at three months after radiotherapy completion. **(Ad)** The enhancing lesion continued to increase in size three months later and was confirmed to represent tumor recurrence after repeat surgery. Images **(Ba–Bd)** demonstrate pseudoprogression. **(Ba)** Pre-operative MRI of a glioblastoma in the insula lobe. **(Bb)** Post-operative MRI at 24 hours after surgery; post-operative blood products are present but there is no contrast enhancement therefore no identifiable residual tumor. **(Bc)** The patient underwent a standard care regimen of radiotherapy and temozolomide. A new rim-enhancing lesion was present on MRI at five months after radiotherapy completion. **(Bd)** Follow-up MRI at monthly intervals showed a gradual reduction in the size of the rim-enhancing lesion without any change in the standard care regimen of radiotherapy and temozolomide or corticosteroid use. The image shown here is the MRI four months later.

Glioblastoma is the most common primary malignant brain tumor with a median 14.6 month overall survival (1). This is in spite of a standard care regimen comprising maximal debulking surgery, followed by radiotherapy with concomitant temozolomide, followed by adjuvant temozolomide. Monitoring biomarkers (2) identify longitudinal change in the growth of tumor or give evidence of response to treatment, with magnetic resonance imaging (MRI) proving particularly useful in this regard. This is due both to the non-invasive nature of MRI, and its ability to capture the entire tumor volume and adjacent tissues, leading to its recommended incorporation into treatment response evaluation guidelines in trials (3, 4). Yet challenges occur when false-positive progressive disease (pseudoprogression) is encountered, which may take place during the 6 month period following the completion of radiotherapy and is manifest as an increase in contrast enhancement on *T*_1_-weighted MRI images, which reflects the non-specific disruption of the blood-brain barrier (**Figure 1**) (5, 6).

Non-specific increased contrast enhancement occurs in approximately 50% of patients undergoing the standard care regimen. There is an approximately equal chance that the tumor may represent pseudoprogression or true progression because pseudoprogression occurs in approximately 10-30% of all patients (7, 8). For more than a decade, researchers have attempted to distinguish pseudoprogression from true progression at the time of increased contrast enhancement because of the substantial potential clinical impact. If there is true progression the treating clinical team typically will initiate a prompt modification in treatment strategy with termination of ineffectual treatment or initiation of second-line surgery or therapies (9). If there is pseudoprogression the treating clinical team typically will continue with the standard care regimen. However, the decision making can only be made retrospectively with current treatment response evaluation guidelines (4). A monitoring biomarker (2) that reliably distinguishes pseudoprogression from true progression at the time of increased contrast enhancement would fully inform the difficult decision contemporaneously.

Under the standard care regimen, pseudoprogression occurs as an early-delayed treatment effect as opposed to radiation necrosis which is a late-delayed radiation effect (10). Radiation necrosis also manifests as non-specific increased contrast enhancement, however, pseudoprogression appears within 6 months of radiotherapy completion whereas radiation necrosis occurs beyond 6 months. Radiation necrosis occurs with an incidence an order of magnitude less than that of pseudoprogression (11). Another difference between the two entities is that much evidence suggests that pseudoprogression is significantly correlated with O^6^-methylguanine DNA methyltransferase (MGMT) promoter methylation. As with pseudoprogression, there is a need to distinguish radiation necrosis from true progression at the time of increased contrast enhancement because, again, there is substantial potential clinical impact. In particular, if there is true progression the treating clinical team typically would initiate second-line surgery or therapies. However, the decision making can only be made retrospectively with current treatment response evaluation guidelines (3). Therefore, a monitoring biomarker (2) that reliably distinguishes radiation necrosis from true progression at the time of increased contrast enhancement would fully inform the treating clinical team’s decision contemporaneously.

Developing monitoring biomarkers to determine treatment response has been the subject of many studies, with many incorporating machine learning (ML). A review of such neuro-oncology studies up to September 2018 showed that the evidence is relatively low level, given that it has usually been obtained in single centers retrospectively and often without hold-out test sets (11, 12). The review findings suggested that those studies taking advantage of enhanced computational processing power to build neuro-oncology monitoring biomarker models, for example deep learning techniques using convolutional neural networks (CNNs), have yet to show benefit compared to ML techniques using explicit feature engineering and less computationally expensive classifiers, for example using support vector machines or even multivariate logistic regressions. Furthermore, studies show that using ML to make neuro-oncology monitoring biomarker models does not appear to be superior to applying traditional statistical methods when analytical validation and diagnostic performance is considered (the fundamental difference between ML and statistics is that statistics determines population inferences from a sample, whereas ML extracts generalizable predictive patterns). Nonetheless, the rapidly evolving discipline of applying radiomic studies to neuro-oncology imaging reflects a recent exponential increase in published studies applying ML to neuroimaging (13), and specifically to neuro-oncology imaging (14). It also mirrors the notable observation that in 2018, arXiv (a repository where computer science papers are self-archived before publication in a peer-reviewed journal) surpassed 100 new ML pre-prints per day (15). Given these developments, there is a need to appraise the evidence of ML applied to monitoring biomarkers determining treatment response since September 2018.

The aim of the study is to systematically review and perform a meta-analysis of diagnostic accuracy of ML-based treatment response monitoring biomarkers for glioblastoma patients using recently published peer-reviewed studies. The study builds on previous work to incorporate the rapidly growing body of knowledge in this field (11, 16), providing promising avenues for further research.

## 2 Materials and Methods

This systematic review and meta-analysis are registered with PROSPERO (CRD42021261965). The review was organized in line with the Preferred Reporting Items for Systematic Reviews and Meta-Analysis: Diagnostic Test Accuracy (PRISMA-DTA) (17) incorporating Cochrane review methodology relating to “developing criteria for including studies” (18), “searching for studies” (19), and “assessing methodological quality” (20).

Pseudoresponse (bevacizumab-related response mimic), an important concern in the United States where it is licensed, was not the focus of the systematic review and meta-analysis.

### 2.1 Search Strategy and Selection Criteria

Recommendations were followed to perform a sensitive search (with low precision), including the incorporation of subject headings with exploded terms, and without any language restrictions (19). Search terms were applied to MEDLINE, EMBASE and the Cochrane Register to capture original research articles published from September 2018 to January 2021 (**Supplementary Table S1**). Pre-prints and non-peer reviewed material were excluded.

#### 2.1.1 Inclusion Criteria

Study participants included were adult glioblastoma patients treated with a standard care regimen (maximal debulking surgery, followed by radiotherapy with concomitant temozolomide, followed by adjuvant temozolomide) who underwent follow-up imaging to determine treatment response status (explicitly, differentiating true progression/recurrence from mimics of progression/recurrence (defined below), and designated as the target condition of the systematic review).

#### 2.1.2 Exclusion Criteria

Studies were excluded if they focused on pediatrics, pseudoresponse, or had no ML algorithm employed in the extraction or selection of features, or in classification/regression.

#### 2.1.3 Index Test and Reference Standard

The ML model determined the treatment response outcome, and was designated as the index test of the systematic review. Either clinicoradiological follow up or histopathology at re-operation or a combination of both, were designated as the reference standard of the systematic review. The bibliography of each included article was checked manually for other relevant studies.

A neuroradiologist, T.C.B., and a data scientist, A.C., with 16 and 2 years, respectively, of experience in neuroimaging applied to neuro-oncology, independently performed the literature search and selection.

### 2.2 Data Extracted and Risk of Bias Assessment

For every study, risk of bias as well as concerns regarding applicability, were assessed by applying QUADAS 2 methodology (21) alongside proformas incorporating items from the Checklist for Artificial Intelligence in Medical Imaging (CLAIM) (22). Data was extracted from published studies to determine: whether the datasets analyzed contained any tumors other than glioblastomas, especially anaplastic astrocytomas and anaplastic oligodendrogliomas; the index test ML algorithm and any cross validation processes; training and hold-out test set information; what reference standard(s) were employed; non-imaging features and MRI sequence(s) included in the analysis.

The appropriateness of reference standard follow-up imaging protocols was reviewed. The handling of confounding factors such as second-line medication therapy, temozolomide cessation, and steroid use were assessed. It was also determined whether the treatment response (target condition) used in the published study was appropriate. Under the standard care regimen, contrast-enhancing lesions enlarging due to pseudoprogression typically occur within 0-6 months after radiotherapy, whereas contrast-enhancing lesions enlarging due to radiation necrosis typically occur beyond this 6 month window, according to the evidence. When “post-treatment related effects” (PTRE) is employed as a term for treatment response outcome, the phenomena of pseudoprogression and radiation necrosis are both included (23, 24). These three terms therefore capture detail regarding the time period when the mimics of progression/recurrence occur. Deviations in the use of the three terms defined here were noted. Data on the length of follow-up imaging after contrast-enhancing lesions enlarged were additionally extracted and evaluated. Clinicoradiological strategies considered optimal in designating outcomes as PTRE or true progression/recurrence included the following: assigning an MRI scan as baseline after radiotherapy (25); excluding outcomes based on *T*_2_-w lesion enlargement (25); permitting a period of 6-month follow up from the first time when contrast-enhancing lesions enlarged; during this 6-month period having two subsequent follow-up scans as opposed to a single short interval “confirmatory” follow-up scan. Two follow-up scans mitigate against some scenarios where the contrast-enhancing lesions due to PTRE continue to enlarge over a short interval, and this continued enlargement is seen at a short interval scan confounding assessment by falsely “confirming” true progression (26, 27). This might be termed an “upslope effect”.

A neuroradiologist (US attending, UK consultant), T.C.B., and a data scientist, A.C., with 16 and 2 years, respectively, of experience in neuroimaging applied to neuro-oncology, independently performed the data extraction and quality assessment. Discrepancies between the two reviewers were considered at a research meeting chaired by a third neuroradiologist (US attending, UK consultant), A.A-B. (8 years experience of neuroimaging applied to neuro-oncology), until a consensus was reached.

### 2.3 Data Synthesis and Statistical Analysis

#### 2.3.1 Performance Accuracy for Individual Studies

Based on the published study data, 2 x 2 contingency tables were made for hold-out test sets from which the principal diagnostic accuracy measures of sensitivity (recall) and specificity were calculated. The area under the receiver operating characteristic curve (ROC-AUC) values and confidence intervals were extracted in studies where these were published. Additional secondary outcome measures of balanced accuracy, precision (positive predictive value) and F1-score were also determined from the contingency tables. In those studies where there was a discrepancy in the principal diagnostic accuracy measures and the accessible published study raw data, this was highlighted. If both internal and external hold-out test sets were published in a study, the principal diagnostic accuracy measures for the external test set alone were calculated. In studies without hold-out test sets, “no test set” was recorded (22) and the training set principal diagnostic accuracy measures from the training set were summarized. The unit of evaluation was per-patient. All test set data included glioblastoma.

#### 2.3.2 Meta-Analysis

The principal diagnostic accuracy measures of sensitivity (recall) and specificity were subject to meta-analysis. We determined two pooled primary measures of accuracy: the true positive rate (sensitivity/recall) and the false negative rate (1-specificity). A bivariate random-effect model (28), which allows for two important circumstances (29–31) (**Supplementary Statistical Information**), was chosen to determine the two pooled primary measures of accuracy. Briefly, the circumstances are first, that the values of the selected principal diagnostic accuracy measures are usually highly related to one another through the cut-off value. With an increase of sensitivity, specificity is likely to decrease and, as a consequence, these two measures are usually negatively correlated. Second, a relatively high level of heterogeneity is commonly observed among the results of diagnostic studies. This is verified in various ways ranging from visual assessment through chi-square based tests to random-intercept models decomposing total variance of results into between- and within- study levels. The bivariate random-effect model not only allows for the simultaneous analysis of diagnostic measures but also addresses their heterogeneity (28). Bivariate joint modelling of the primary measures of accuracy assumes that the logits of these quantities follow a bivariate normal distribution and allows for a non-zero correlation. Based on this assumption, a linear random-effect model is applied to the data and estimates of mean true positive rate (sensitivity) and false positive rate (1-specificity), along with their variances and correlation between them, can be obtained. The pooled estimates of true positive rate and false positive rate are initially estimated on the logit scale (**Supplementary Statistical Information**). To be interpretable they require transformation back to the original probability scale (ranging within 0-1 limits).

The parameters of this model also allowed us to plot the summary ROC (SROC) curve and determine the summary ROC-AUC. Using a resampling approach (32), the model estimates were also used to derive the pooled measures of balanced accuracy as well as the positive and negative likelihood ratios and the diagnostic odds ratio.

The meta-analysis was conducted by a statistician, M.G., with 15 years of relevant experience. All the statistical analyses were performed in R (v 3.6.1). The R package mada (v 0.5.10) (33) was used for the bivariate model. Since some of the 2 x 2 contingency table input cell values (true positive, false positive, false negative, true negative) derived from the individual studies contained zeros, a continuity correction (0.5) was applied.

### 2.4 Prognostic Biomarkers Predicting Future Treatment Response

Most studies of prognostic imaging biomarkers in glioblastoma predict the outcome measure of overall survival using baseline images. Nonetheless, we found a small group of studies using ML models that predicted the outcome measure of future treatment response using baseline images. The studies were examined using identical methodology to that applied to monitoring biomarkers.

## 3 Results

### 3.1 Characteristics and Bias Assessment of Studies Included

**Figure 2:**
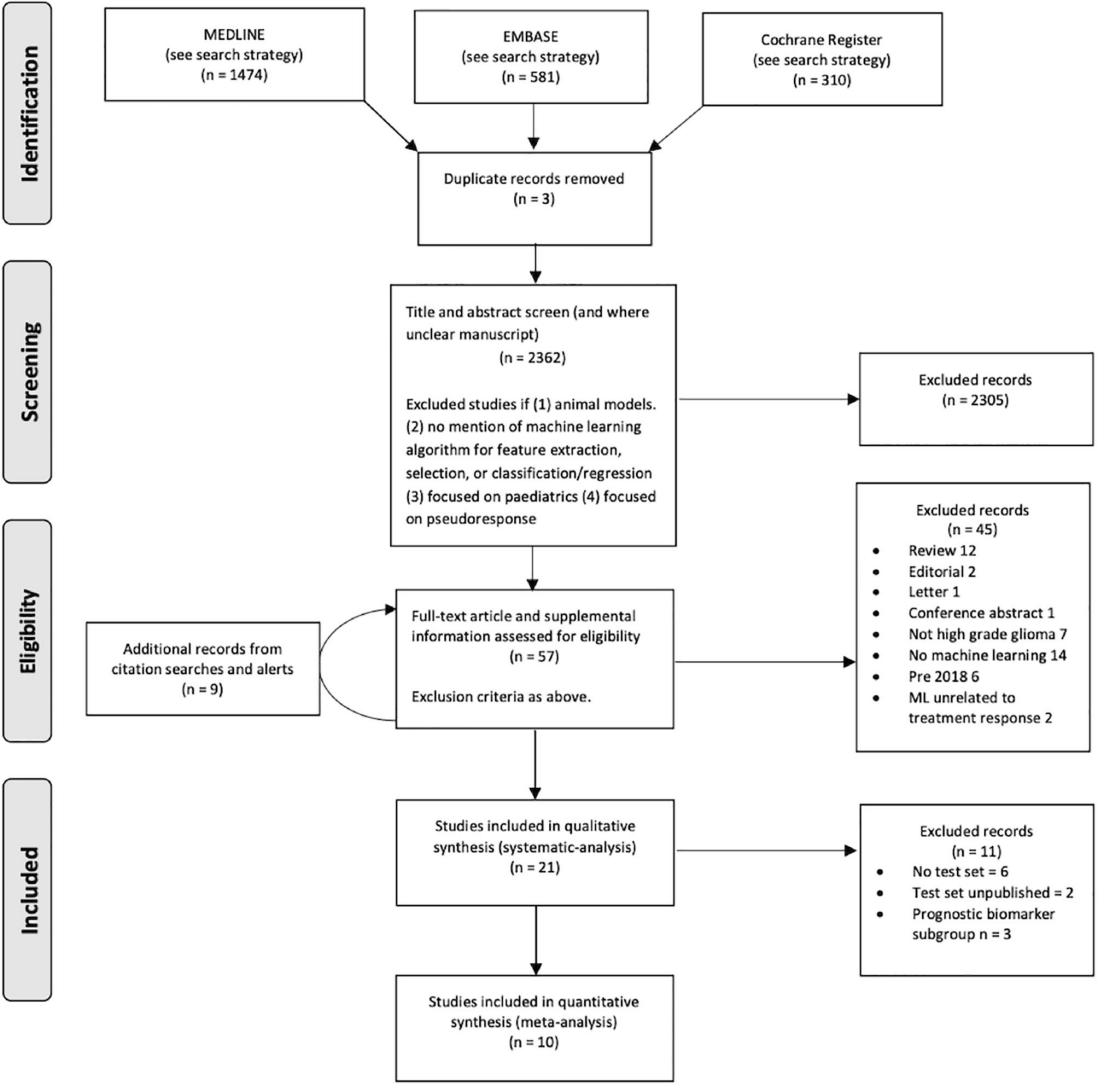
Flow diagram of search strategy.

In all, 2362 citations fulfilled the search criteria of which the full text of 57 potentially eligible articles were reviewed (**Figure 2**). Twenty-one studies from September 2018 to January 2021 (including the publication of “online first” articles prior to September 2018) were included, 19 of which were retrospective. The total number of patients in the training sets were 1335 and in the test set 384. The characteristics of the 18 monitoring biomarker studies are presented in **Table 1** and the characteristics of the 3 studies that applied the ML models to serve as prognostic biomarkers to predict future treatment response using baseline images (or genomic alterations) are presented in **Table 2**.

**Table 1:** Studies using machine learning in the development of glioblastoma monitoring biomarkers.

Author	Target condition	Reference standard	Dataset(s)	Available demographic information	Methodology	Features selected	Test set performance
^a^Kim J.Y. et al. (34)	Early true progression or Early pseudoprogression	Mixture of histopathology and imaging follow up	Training = 61 Testing = 34 *T*_1_ C, FLAIR, DWI, DSC	Training = age mean ± SD (range) 58 ± 11 (34–83) male 38 (62%) Testing = age mean ± SD 62 ± 12 male 25 (74%) Data from Korea	Retrospective 2 centers: 1 train & 1 external test set. LASSO feature selection with 10-fold CV Linear generalized model	First-order, volume/shape, Second-order (texture), wavelet. ADC & CBV parameters included.	Recall 0.71 Specificity 0.90 Precision 0.83 BA 0.81 F1 0.77 AUC 0.85 (CI 0.71 – 0.99)
Kim J.Y. et al. (35)	Early true progression or Early pseudoprogression	Mixture of histopathology and imaging follow up	Training = 59 Testing = 24 *T*_1_ C, FLAIR, DTI, DSC	Training = age mean ± SD 61 ± 11 male 37 (63%) Testing = age mean ± SD 59 ± 12 male 9 (38%) Data from Korea	Retrospective 1 center LASSO feature selection with 10-fold CV Linear generalized model	First-order, Second-order (texture), wavelet. FA & CBV parameters included.	Recall 0.80 Specificity 0.63 Precision 0.36 BA 0.72 F1 0.50 AUC 0.67 (0.40 – 0.94)
Bacchi S. et al. (36)	True progression or PTRE (HGG)	Histopathology for progression and imaging follow up for pseudoprogression	Training = 44 Testing = 11 *T*_1_ C, FLAIR, DWI	Combined = age mean ± SD 56 ± 10 male 26 (47%) Data from Australia	Retrospective 1 center 3D CNN & 5-fold CV	CNN. FLAIR & DWI parameters	Recall 1.00 Specificity 0.60 Precision 0.75 BA 0.80 F1 0.86 AUC 0.80
Elshafeey N. et al. (37)	True progression or ^b^PTRE	Histopathology	Training = 98 Testing = 7 DSC, DCE	Training = age mean ± SD 50 ± 13 male 14 (58%) No testing demographic information Data from USA	Retrospective 3 centers mRMR feature selection. 1 test. 1) decision tree algorithm C5.0 2) SVM including LOO and 10-fold CV	K_trans_ & CBV parameters	Insufficient published data to determine diagnostic performance (CV training results available recall 0.91; specificity 0.88)
Verma G. et al. (38)	True progression or Pseudoprogression	Mixture of histopathology and imaging follow up	Training = 27 3D-EPSI	Training = age mean ± SD 64 ± 10 male 14 (52%) Data from USA	Retrospective 1 center Multivariate logistic regression LOOCV	Cho/NAA & Cho/Cr	No test set (CV training results available recall 0.94; specificity 0.87)
Ismail M. et al. (39)	True progression or Pseudoprogression	Mixture of histopathology and imaging follow up	Training = 59 Testing = 46 *T*_1_ C, *T*_2_/ FLAIR	Training = age mean(range) 61 (26–74) male 39 (66%) Testing = age mean (range) 56 (25–76) male 30 (65%) Data from USA	Retrospective 2 centers: 1 train & 1 external test set. SVM & 4-fold CV	Global & curvature shape	Recall 1.00 Specificity 0.67 Precision 0.88 BA 0.83 F1 0.94
^a^Bani-Sadr A. et al. (40)	True progression or Pseudoprogression	Mixture of histopathology and imaging follow up	Training = 52 Testing = 24 *T*_1_ C, FLAIR MGMT promoter status	Combined = age mean ± SD 58 ± 11 male 45 (59%) Data from France	Retrospective 1 center Random Forest.	Second-order features +/- MGMT promoter status	Recall 0.94 (0.71 - 1.00) Specificity 0.38 (0.09 - 0.76) Precision 0.36 BA 0.66 F1 0.84 AUC 0.77 & non-MRI: Recall 0.80 (0.56 - 0.94) Specificity 0.75 (0.19 - 0.99) Precision 0.86 BA 0.74 F1 0.83 AUC 0.85
Gao X.Y. et al. (41)	True progression or PTRE (HGG)	Mixture of histopathology and imaging follow up	Training = 34 Testing = 15 (per lesion) *T*_1_ C, FLAIR	Combined = age mean ± SD 51 ± 11 male 14 (36%) (per patient) Data from China	Retrospective 2 centers SVM & 5-fold CV	*T*_1_ C, FLAIR subtraction map parameters	Recall 1.00 Specificity 0.90 Precision 0.83 BA 0.95 F1 0.91 AUC 0.94 (0.78 – 1.00)
Jang B-S. et al. (42)	True progression or Pseudoprogression	Mixture of histopathology and imaging follow up	Training = 59 Testing = 19 *T*_1_ C & clinical features & IDH/MGMT promoter status	Training = age median (range) 56 (22–77) male 41 (70%) Testing = age mean ± SD 53 (28–75) male 10 (53%) Data from Korea	Retrospective 2 centers 1 train & 1 external test set. CNN LSTM & 10-fold CV (compared to Random Forest)	CNN *T*_1_ C parameters +/- Age; Gender; MGMT status; IDH mutation; radiotherapy dose and fractions; follow-up interval	Recall 0.64 Specificity 0.50 Precision 0.64 BA 0.57 F1 0.63 AUC 0.69 & non-MRI: Recall 0.72 Specificity 0.75 Precision 0.80 BA 0.74 F1 0.76 AUC 0.83
Li M. et al. (43)	True progression or ^b^PTRE	Imaging follow up	Training = 84 DTI	No demographic information Data from USA	Retrospective. 1 center DC-AL GAN CNN with SVM including 5 and 10 and 20-fold CV (compared to DCGAN, VGG, ResNet, and DenseNet)	CNN. DTI	No test set (CV training results only available: Recall 0.98 Specificity 0.88 AUC 0.95)
Akbari H. et al. (44)	True progression or Pseudoprogression	Histopathology	Training = 40 Testing = 23 Testing = 20 *T*_1_ C, *T*_2_/FLAIR, DTI, DSC, DCE	Combined internal = age mean (range) 57 (33–82) male 38 (60%) No external demographic information Data from USA	Retrospective 2 centers. 1 train & test. 1 external test set. imagenet_vgg_f CNN SVM & LOOCV	First-order, second-order (texture). CBV, PH, TR, *T*_1_ C, *T*_2_/FLAIR parameters included.	Recall 0.70 Specificity 0.80 Precision 0.78 BA 0.75 F1 0.74 AUC 0.80
Li X. et al. (45)	Early True progression or early pseudoprogression (HGG)	Mixture of histopathology and imaging follow up	Training = 362 *T*_1_ C, *T*_2_, multi-voxel & single-voxel 1H-MRS, ASL	Training = age mean (range) 50 (19–70) male 218 (60%) Data from China	Retrospective Gabor dictionary and sparse representation classifier (SRC)	Sparse representations	No test set (CV training results only available: Recall 0.97 Specificity 0.83)
Manning P et al. (46)	True progression or pseudoprogression	Mixture of histopathology and imaging follow up	Training = 32 DSC, ASL	Training = age mean ± SD 56 ± 13 male 22 (69%) Data from USA	Retrospective 1 center Linear discriminant analysis & LOOCV	CBF and CBV parameters included.	No test set (CV training results only available: Recall 0.92 Specificity 0.86 AUC 0.95)
Park J.E. et al., 2020 (47)	Early True progression or early pseudoprogression	Mixture of histopathology and imaging follow up	Training = 53 Testing = 33 *T*_1_ C	Training = age mean ± SD 56 ± 11 male 31 (59%) Testing = age mean ± SD 62 ± 12 male 25 (76%) Data from Korea	Retrospective 2 centers. 1 train & test. 1 external test set. Random Forest feature selection with 10-fold CV (Automated segmentation)	First-order, volume/shape, Second-order (texture), wavelet parameters included.	Recall 0.61 Specificity 0.47 Precision 0.58 BA 0.54 F1 0.59 AUC 0.65 (0.46 – 0.84)
Lee J. et al. (48)	True progression or ^b^PTRE (HGG)	Histopathology	Training = 43 *T*_1,_ *T*_1_ C, *T*_2,_ FLAIR, (subtractions: *T*_1_ C - *T*_1_, *T*_2-_ FLAIR) ADC parameters.	Training =age mean ± SD (range) 52 ± 13 (16–74) male 24 (56%) Data from USA	Retrospective 1 center CNN-LSTM. 3-fold CV	CNN-LSTM parameters.	No test set (CV training results only available: AUC 0.81 (0.72 - 0.88))
Kebir S. et al. (49)	True progression or ^b^PTRE	Imaging follow up	Training = 30 Testing = 14 O-(2[^18^F]-fluoroethyl)-L-tyrosine (FET)	Combined = age mean ± SD (range) 57 ± 11 (34-79) male 34 (77%) Data from Germany	Retrospective 1 center Linear discriminant analysis. 3-fold CV	TBR_mean_ TBR_max_ TTP_min_ parameters.	Recall 1.00 Specificity 0.80 Precision 0.90 BA 0.92 F1 0.95 AUC 0.93 (0.78 - 1.00)
Cluceru J. et al. (50)	Early True progression or early pseudoprogression (HGG)	Histopathology	Training = 139 DSC, MRSI, DWI, DTI	Training = age median (range) 52 (21–84) Male 83 (60%) Data from USA Ethnicity: White 112 (80%) American Indian 1 (1%) Asian 6 (4%( Pacific Islander 2 (1%) Other 18 (13%)	Retrospective 1 center Multivariate logistic regression. 5-fold CV	Cho, Cho/Cr, Cho/NAA & CBV parameters.	No test set (CV training results only available: Recall 0.65 (0.33 - 0.96); Specificity 0.62 (0.21 - 1.00) AUC 0.69 (0.51 - 0.87))
Jang B.S. et al. (51)	True progression or ^b^PTRE	Mixture of histopathology and imaging follow up (including PET)	(i) (trained model = 78) testing = 104 (ii) all training = 182 *T*_1_ C & clinical, molecular, timings, radiotherapy data	Testing = age median (range) 55 (25-76) male 59 (67%) Data from Korea	Retrospective (i) 6 centers 1 external test set. CNN LSTM (ii) 7 centers 1 training set CNN LSTM & 10-fold CV	CNN *T*_1_ C parameters and Age; Gender; MGMT status; IDH mutation; radiotherapy dose and fractions; follow-up interval	(i) Insufficient published data to determine diagnostic performance (ii) No test set (CV training results available AUPRC 0.87)

a Within publication some data appears mathematically discrepant.

b Within publication discrepant or unclear information (e.g. interval after radiotherapy).

Unless otherwise stated, glioblastoma alone was analyzed.

PTRE, post-treatment related effects; HGG, high-grade glioma.

MRI sequences: T_1_ C, postcontrast T_1_-weighted; T_2_, T_2_-weighted; FLAIR, fluid-attenuated inversion recovery; DSC, dynamic susceptibility-weighted; DCE, dynamic contrast-enhanced; DWI, diffusion-weighted imaging; DTI, diffusor tensor imaging; ASL, arterial spin labelling; MRI parameters: ADC, apparent diffusion coefficient; FA, fractional anisotropy; TR, trace (DTI); CBV, cerebral blood volume; PH, peak height; K_trans_, volume transfer constant.

Magnetic resonance spectroscopy: 1H-MRS, 1H-magnetic resonance spectroscopy; 3D-EPSI, 3D echo planar spectroscopic imaging.

1H-MRS parameters: Cr, creatine; Cho, choline; NAA, N-acetyl aspartate.

Nuclear medicine: TBR, tumor-to-brain ratio; TTP, time-to-peak.

Molecular markers: MGMT, O6-methylguanine-DNA methyltransferase; IDH, isocitrate dehydrogenase.

Machine learning methodology: CV, cross validation; LOOCV, leave-one-out cross validation; SVM, support vector machine; CNN, convolutional neural network; LASSO, least absolute shrinkage and selection operator; LSTM, long short-term memory; mRMR, minimum redundancy and maximum relevance; VGG, Visual Geometry Group (algorithm); DCGAN, deep convolutional generative adversarial network; DC-AL GAN, DCGAN with AlexNet.

Statistical measures: CI, confidence intervals; BA, balanced accuracy; AUC, area under the receiver operator characteristic curve; AUPRC, area under the precision-recall curve.

**Table 2:** Studies applying machine learning models to baseline MRI images (or genomic signatures) to operate as glioblastoma prognostic biomarkers to predict future treatment response.

Author	Target condition	Reference standard	Dataset(s)	Available demographic information	Methodology	Features selected	Test set performance
Wang S. et al. (52)	True progression or pseudoprogression (immunotherapy for EGFRvIII mutation) Baseline prediction	Histopathology	model testing set = 10 DTI, DSC and 3D-EPSI	Testing = age mean (range) 55 (45-77) ± 8 male 4 (40%) Data from USA	Prospective. 1 center. Multivariate logistic regression.	CL, CBV, FA parameters	Insufficient data to determine per patient diagnostic performance (per lesion results only available: Recall = 0.86 Specificity = 0.60)
Yang K. et al. (53)	True progression or not (stable disease, partial & complete response & pseudoprogression) Baseline prediction	Imaging follow up	Training = 49 Genomic alterations	Training = age median (range) 57 (22-82) male 30 (61%) Data from Korea	Retrospective. 1 center. Analysis including Gene Set Enrichment Analysis (GSEA).	Genomic alterations including CDKN2A and EGFR mutations	No test set (Insufficient data to determine per patient diagnostic performance. From training dataset: 1-year PFS for responder 45%; non-responder 0%)
Lundemann M. et al. (54)	Early recurrence or not (voxel-wise) Baseline prediction	Mixture of histopathology and imaging follow up	Training = 10 18F-FET PET/CT; 18F-FDG PET/MRI; *T*_1_ C; *T*_2_/FLAIR; DTI; DCE	Training = age mean (range) 54 (40-71) male 7 (78%) Data from Denmark	Prospective. 1 center. Multivariate logistic regression LOOCV.	FET; FDG; MD, FA; F, Vb, Ve, Ki, and MTT parameters.	No test set (Insufficient data to determine per patient diagnostic performance. From training dataset: Voxel-wise recurrence probability AUC 0.77)

EGFR, epidermal growth factor receptor; EGFRvIII, epidermal growth factor receptor variant III; CDKN2A, cyclin-dependent kinase Inhibitor 2A.

MRI sequences: T_1_ C, post-contrast T_1_-weighted; T_2_, T_2_-weighted; FLAIR, fluid-attenuated inversion recovery; DSC, dynamic susceptibility-weighted; DCE, dynamic contrast-enhanced; DTI, diffusor tensor imaging.

Other imaging techniques: 3D-EPSI, 3D echo planar spectroscopic imaging; PET/CT, positron emission tomography and computed tomography; PET/MRI, positron emission tomography and magnetic resonance imaging; 18F-FDG, [18F]-fluorodeoxyglucose; 18F-FET, [18F]-fluoroethyl-L-tyrosine.

MRI parameters: FA, fractional anisotropy; MD, mean diffusivity; CL, linear anisotropy; CBV, cerebral blood volume; MTT, mean transit time; F, blood flow; Ve, extra-vascular extra-cellular blood volume; Vb, vascular blood volume; Ki, vascular permeability.

Statistical and machine learning methodology: LOOCV, leave-one-out cross validation; AUC, area under the receiver operator characteristic curve; PFS, progression free survival.

#### 3.1.1 Treatment Response Target Conditions

The treatment response target conditions varied between studies (**Table 1**). Around a quarter of studies (5/18, 28%) designated only 0-12 weeks after radiotherapy as the time period when pseudoprogression appears – as opposed to the entire 6-month time period when pseudoprogression might occur. A third of studies (6/18, 33%) assigned PTRE as the target condition. No study assigned radiation necrosis alone as the target condition. Five studies in the systematic review (5/18, 28%) included grade 3 gliomas. Only two of these five studies employed test sets; the test set in one study did not contain any grade 3 gliomas and the number in the test set in the other study was unclear although the number was small (14% grade 3 in combined training and test datasets). Therefore, as a minimum, all but one test set in the systematic review and meta-analysis contained only glioblastoma, the previous equivalent of glioma grade 4 according to c-IMPACT classification (“glioblastomas, IDH-wildtype” or “astrocytoma, IDH-mutant, grade 4”) (55).

#### 3.1.2 Reference Standards: Clinicoradiological Follow-Up and Histopathology Obtained at Re-Operation

The majority of studies (13/18, 67%) employed a combination of clinicoradiological follow up and histopathology at re-operation, to distinguish true progression from a mimic. A few individual studies employed one reference standard for one decision (true progression) and another reference standard for the alternative decision (mimic); this and other idiosyncratic rules led to a high risk of bias in terms of the reference standard used, as well as how patients were selected, in several studies.

#### 3.1.3 Selected Features

Most studies only analyzed imaging features alone (15/18, 83%) whereas the remainder incorporated additional non-imaging features. A third of studies (6/18, 33%) used deep learning methodology to derive features (specifically, convolutional neural networks).

#### 3.1.4 Test Sets

A third of studies did not have hold-out test sets (6/18, 33%) and instead the performance accuracy was determined using training data through cross-validation (**Table 1**). Therefore, there was a high risk of bias for the index test used in these six studies. A third of studies had external hold-out test sets (6/18, 33%). The ranges of mean diagnostic accuracy measures in these six studies were: recall (sensitivity) = 0.61-1.00; specificity = 0.47-0.90; precision (positive predictive value) = 0.58-0.88; balanced accuracy = 0.54-0.83; F1 score = 0.59-0.94; ROC-AUC = 0.65-0.85.

#### 3.1.5 Bias Assessment and Concerns Regarding Applicability Summary

The risk of bias evaluation for each study was summarized (**Supplementary Figure S1**). All or most studies were assigned to the highest class for risk of bias in terms of the reference standard (18/18, 100%) and patient selection (15/18, 83%) QUADAS 2 categories respectively. A third or nearly a half of studies were either in the highest class for risk of bias or the risk was unclear in terms of flow and timing (6/18, 33%) and the index test (8/28, 44%) QUADAS 2 categories respectively. The results from the “concerns regarding applicability” evaluation largely mirrored the results of the risk of bias evaluation.

#### 3.1.6 Prognostic Biomarkers Predicting Future Treatment Response (Subgroup)

There were two studies which were prospective, both of which had a small sample size (n = 10); the third study in this subgroup was retrospective. One study applied genomic alterations alone as features to predict future MRI treatment response. All studies (3/3, 100%) were in the highest class for risk of bias in terms of the reference standard, patient selection and index test QUADAS 2 categories (**Supplementary Figure S2**). In terms of “concerns regarding applicability” evaluation, the results mirrored the risk of bias evaluation exactly. Diagnostic accuracy measures could not be calculated because of study design. Design constraints included units of assessment in one study being per-lesion whilst another was per-voxel. One study also incorporated a prognostic metric of 1-year progression free survival for the predicted treatment response target condition. Overall, the studies are best considered as proof of concept. Overall, there was insufficient data to perform a subgroup meta-analysis.

### 3.2 Results of Meta-Analysis

**Figure 3:**
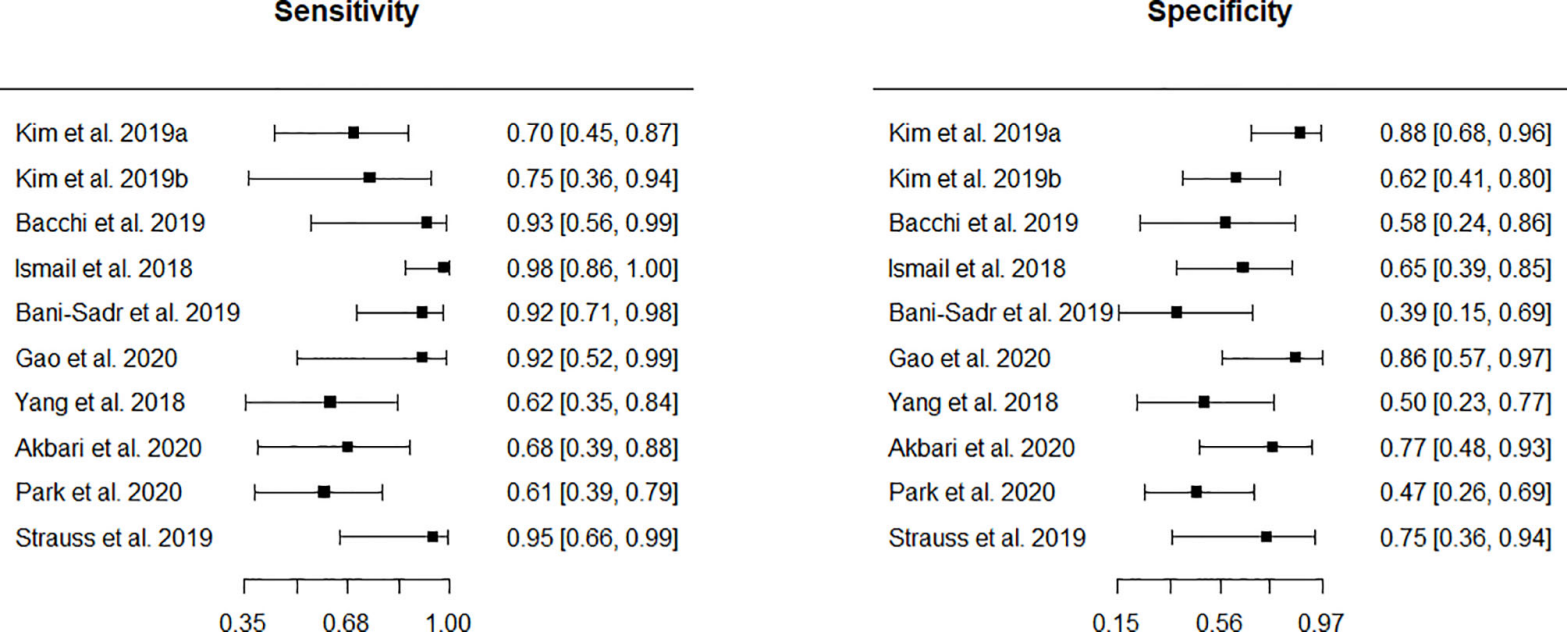
Forest plots showing sensitivity and specificity.

Eleven studies appeared eligible for inclusion in a meta-analysis of monitoring biomarker studies as there was information regarding internal or external hold-out test set data. However, one test was ineligible (n < 10; 3 cells in the 2 x 2 contingency table n = 0). Ten (10/18, 56%) remaining studies were subject to further analyses. Forest plots of sensitivity and specificity (**Figure 3**) graphically showed a high level of heterogeneity. Also, chi-square tests were applied separately to both primary measures. The p values resulting from these tests were 0.017 and 0.110 for sensitivities and specificities, respectively thus indicating the significant heterogeneity. This supported the choice of the bivariate random-effect model. The pooled true positive rate (sensitivity) = 0.769 (0.649 - 0.858) and the pooled false positive rate (1-specificity) = 0.352 (0.251 - 0.468).

**Figure 4:**
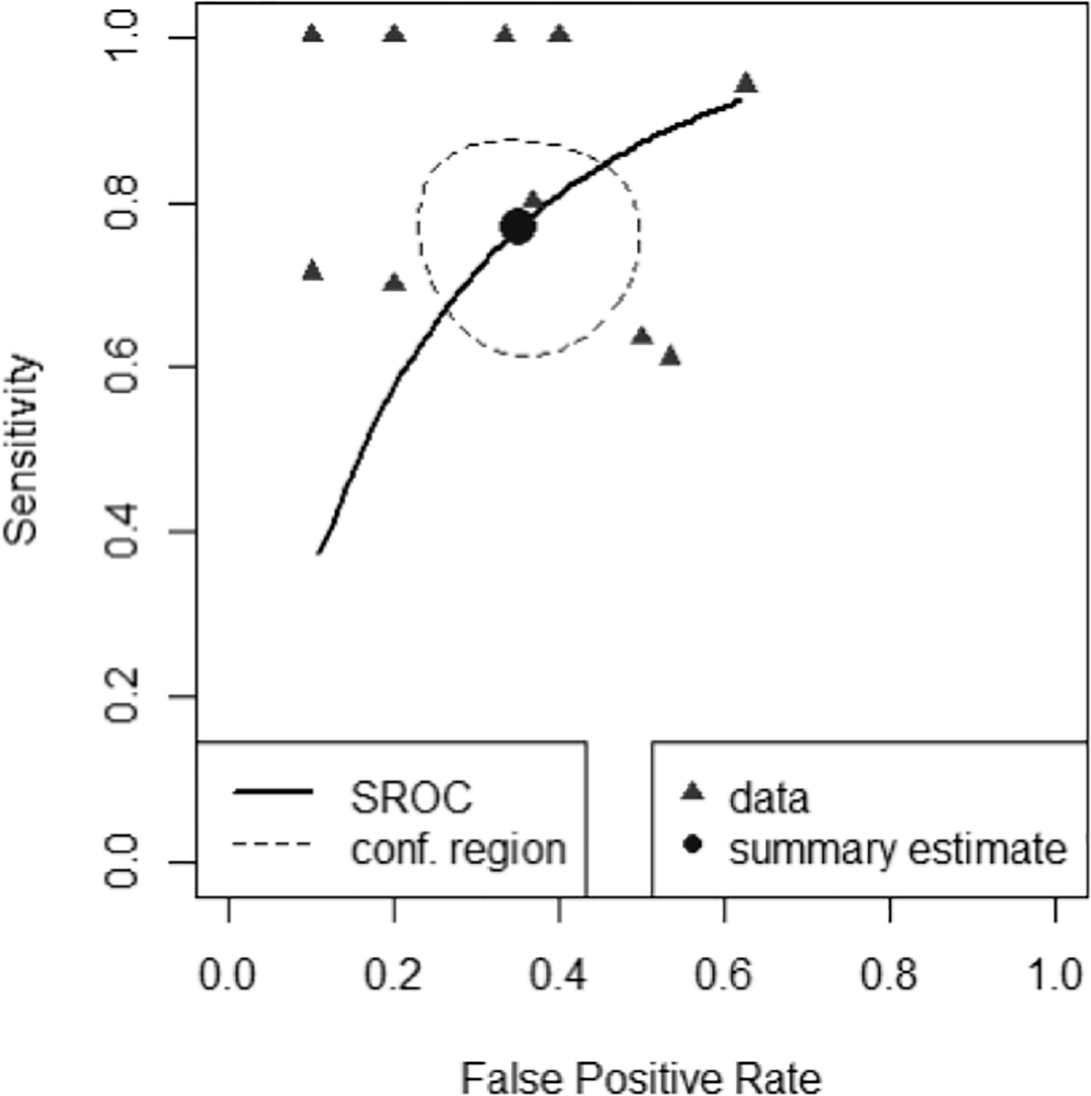
Summary Receiver Operator Characteristic Curve (SROC) of diagnostic performance accuracy. The summary point estimate and surrounding 95% confidence region is shown.

A scatter plot of false positive rates (1-specificity) and true positive rates (sensitivity) shown in **Figure 4** demonstrates individual ROC point estimates and a summary ROC (SROC) curve giving summary ROC-AUC = 0.765.

The derived pooled measures of balanced accuracy = 0.706 (0.623-0.779); positive likelihood ratio = 2.220 (1.560-3.140); negative likelihood ratio = 0.366 (0.213- 0.572); diagnostic odds ratio = 6.670 (2.800-13.500).

## 4 Discussion

### 4.1 Summary of Findings

To date, available evidence is relatively low level (12) for determining the diagnostic accuracy of ML-based glioblastoma treatment response monitoring biomarkers in adults. The available evidence is subject to a number of limitations because recent studies are at a high risk of bias and there are concerns about its applicability, especially when determining the status of response to treatment using the reference standards of follow-up imaging or pathology at re-operation. There are similar and associated concerns regarding the selection of study patients. A third of the studies did not include any type of hold-out test set. Most of the studies employed classic ML approaches based on radiomic features. A third of studies employed deep learning methodologies.

### 4.2 Limitations

#### 4.2.1 Studies Assessed

Limitations encompassed three main areas. First, the reference standards used in all studies resulted in a high risk of bias and concerns about applicability. With the exception of the prognostic biomarker subgroup of studies, all the studies were retrospective, which increased the risk of confounding. Confounding factors, in relation to imaging follow-up and pathology at re-operation reference standards, were second-line drug therapy and cessation of temozolomide, all of which were rarely considered. Likewise, the use of corticosteroids was rarely considered despite being a confounding factor in relation to the imaging follow-up reference standard. If unaccounted for, an increase in corticosteroid dose may cause false negative treatment response. Some authors provided a statement within their methodology that they followed RANO guidelines (4) which if followed meticulously would surmount some of these clinicoradiological limitations, such as the use of corticosteroids which is integrated with the imaging assessment. One limitation in using the RANO guidelines, however, is that in some scenarios the contrast-enhancing lesions due to PTRE continue to enlarge over a short interval, confounding assessment by falsely confirming true progression if continued enlargement is seen at a second short interval scan; RANO guidelines do not account for this upslope effect (26, 27).

Second, patient selection was problematic and is associated with confounding. For example, patients receiving second-line drug therapy should have been excluded as response assessment may be altered. It is also noteworthy that astrocytoma, IDH-mutant, grade 4 are biologically and prognostically distinct from glioblastomas, IDH-wildtype (55). Variable proportions in individual studies introduces between-study heterogeneity and therefore this is a source of potential confounding when comparing or pooling data. Nonetheless, it is acknowledged that for grade 4 tumors, IDH-mutants have a prevalence an order of magnitude less than IDH-wildtype, likely limiting the impact of such confounding.

Third, hold-out test sets should be used for diagnostic accuracy assessment in ML studies (22) as it is a simple demonstration as to whether the trained model overfits data; nonetheless more than a third of studies did not use either an internal or external hold-out test set. Nonetheless, six studies did use external hold-out tests which might be considered optimal practice for determining generalizability.

#### 4.2.2 Review Process

Imaging reference standards, especially RANO trial guidelines (4) and later iterations (25), are rarely applied correctly and are themselves confounded (56). Because tumors have a variety of shapes, may have an outline that is difficult to delineate, and may be located only within the cavity rim, it can be challenging to perform seemingly simple size measurements (11). For example, large, cyst-like glioblastomas may be “non-measurable” unless a solid nodular component of the rim fulfils the “measurable” criteria.

As well as the scenario described above highlighting the upslope effect of PTRE (26, 27), another limitation of RANO is a failure to acknowledge that pseudoprogression appears over a 6-month period rather than a 3-month period (although it is accepted that even a 6 month cut-off is arbitrary) (26). Follow-up imaging of adequate duration is therefore required in study design. This leads to a further limitation of this or other systematic reviews – it is extremely difficult to design studies with enough nuance to be at low risk of bias in regards to the reference standard.

Another limitation of this systematic review is that pathology at re-operation, where used as a reference standard, is typically not an entirely reliable reference standard for two reasons (57). First, there is the potential for biopsy sampling bias because the entire enhancing tissue may represent an admixture of PTRE and tumor (58). Second, there is a lack of pathological standardization causing a variety of inter-observer diagnostic interpretations given the background of extensive post-therapy related changes (59). Nonetheless, in the absence of more reliable available reference standards at re-operation, it was pragmatically included as an acceptable reference standard. Additionally, according to many authors, it is closer to being a more accurate reference standard compared to follow-up imaging.

Publication bias may also have affected the range of diagnostic accuracy of the monitoring biomarkers included in this systematic review and meta-analysis. Related to this, the exclusion of pre-prints and non-peer reviewed material may exacerbate publication bias. In particular, given that some in the data science community may not submit their work in peer-reviewed journals as peer review is relatively slow compared to the speed at which data science develops, it is plausible that publication bias relates to the make-up of the researcher team. For example, more clinically-orientated teams may be more inclined to publish in a peer reviewed journal compared to more data science-orientated teams.

### 4.3 Explanation of the Results in the Context of Other Published Evidence

After treatment, “monitoring biomarkers” are measured serially to detect change in the extent of tumor infiltration or to provide evidence of response to treatment (2). In nearly all glioblastomas the integrity of the blood brain barrier is disrupted and MRI is used to take advantage of this. Following intravenous administration of gadolinium-based contrast agents, the hydrophilic contrast molecules diffuse from the vessel lumen and accumulate in the extravascular extracellular space, manifesting on *T*_1_-weighted sequences as contrast-enhancing hyperintense regions (60). Subsequently, MRI has been incorporated into recommendations for determining response to treatment in trials (4). In these recommendations, treatment response assessment is based on simple linear metrics of contrast-enhancing tumor, specifically, the product of maximal perpendicular cross-sectional dimensions in “measurable” lesions defined as > 10 mm in all perpendicular dimensions. The recommendations are based on expert opinion informed by observational studies and derived from the biologically plausible assumption that an increase in the size of a tumor identifies disease progression, potentially resulting in a lead time improvement for therapeutic intervention before the tumor becomes clinically apparent (61). The rationale is that there may be advantages in altering management early on before the onset of irreversible disability or the tumor extent precludes intervention. Justification for enhancement as a proxy for tumor has been inferred from data showing that the size of the enhancing region and extent of resection of the enhancing region are “prognostic biomarkers” (2) at both initial presentation and confirmed recurrence (62–64).

The trial assessment recommendations, incorporated in a less stringent form during routine clinical assessment (65), allow for an early change in treatment strategy (9). However, there are important challenges using conventional structural MRI protocols.

First, treatment response assessment typically is made in a retrospective manner as confirmatory imaging is required to demonstrate a sustained increase or a sustained decrease in enhancing volume. This leads to a delay in diagnosis.

Second, contrast enhancement is biologically non-specific, which can result in false negative, false positive, and indeterminate outcomes, especially in regards to the post-treatment related pseudophenomena observed in glioblastoma patients (61). Pseudoprogression is an early post-treatment related effect characteristically appearing within 6 months of glioblastoma patients completing radiotherapy and concomitant temozolomide, whereas pseudoresponse (not examined in this systematic review) appears after patients have been treated with anti-angiogenic agents such as bevacizumab. False-negative treatment response and false-positive progression appear as a decrease or an increase in the volume of MRI contrast enhancement, respectively. Delayed post-treatment related effects caused by radiation necrosis similarly appear as an increase in volume of MRI contrast enhancement, again potentially causing false-positive progression. A different scenario where contrast enhancement is biologically non-specific includes post-operative peritumoral parenchymal enhancement after operative “tissue handling”; or after operative infarction.

Conventional structural MRI protocols are therefore limited and contemporaneous, accurate and reliable monitoring biomarkers are required for glioblastoma treatment response assessment. Three potential solutions are highlighted here:

First, an emerging alternative approach is to harness the potential value of circulating biomarkers (including circulating tumor cells, exosomes, and microRNAs) to monitor disease progression in glioma patients (66). However, as with any potential monitoring blood or cerebral spinal fluid biomarker, potential use requires further evaluation and validation in large scale prospective studies before implementation into standard clinical practice can be envisaged.

Second, another promising approach is to use advanced imaging techniques (67). The last three decades have seen considerable technical developments in MRI (for example, those related to perfusion, permeability and diffusion), 1H-MR spectroscopic imaging, and positron emission tomography (for example using radiolabelled amino acids). A meta-analysis of 28 perfusion and permeability imaging studies showed that the pooled sensitivities and specificities of each study’s best performing parameter were 90% and 88% (95% confidence interval (CI), 0.85 - 0.94; 0.83 - 0.92) and 89% and 85% (95% CI, 0.78 - 0.96; 0.77 - 0.91) for dynamic susceptibility-weighted (DSC) and dynamic contrast-enhanced (DCE) MRI, respectively (68). Clinical translation is far from ubiquitous (65) reflecting that further investigation and consensus standardization is required before implementing any particular widespread quantitative strategy (68). Indeed, advanced imaging is not yet recommended for determining treatment response in trials (4), and there is a lack of evidence that using advanced MRI techniques leads to a reduction in morbidity or mortality (61). However, compared to ML where accuracy-driven performance metrics have resulted in increasingly opaque models, particularly when using structural images, the underlying biological processes relating to advanced imaging appear to be well understood whilst also demonstrating high performance accuracy.

A third approach is to use ML, whether applied to conventional structural MRI, advanced imaging techniques or a combination of both imaging and non-imaging features. Indeed, an advantage of machine learning applied to MRI is that wide data can be handled relatively easily (11) which might allow the wide spectrum of signatures from multiparametric advanced MRI to be captured together to improve performance accuracy. However, a disadvantage when compared to a single modality approach is that combinations of outputs from individual modalities that are without frameworks for technical and clinical use, might compound inter-center variability and reduce generalizability considerably. The advantages and disadvantages of using ML-based monitoring biomarkers for glioblastoma treatment response assessment have been described recently (summarised in **Table 3**) (61). However, a number of factors demonstrate that only limited conclusions on performance can be drawn from recent studies in our systematic review. These include the high risk of bias and concerns about applicability in study designs, the small number of patients analysed in ML studies, and the low level of evidence of the monitoring biomarker studies given their retrospective nature.

**Table 3:** Advantages and disadvantages of using ML-based monitoring biomarkers for glioblastoma treatment response assessment (61).

Advantages	Disadvantages
Using ML requires less formal statistical training given the huge developments in software (69), and the programming expertise for researchers has now been transformatively reduced, enabled by standardized implementations of open source software (70, 71).	The clinical context may not be represented with a decreased ability to perform holistic evaluations of patients, with loss of valuable and irreducible aspects of the human experience such as psychological, relational, social, and organizational issues (72).
Wide data can be handled relatively easily (11) and ML can be applied to conventional structural MRI, advanced imaging techniques or a combination of both imaging and non-imaging features.	Linking the empirical data to a categorical analysis can neglect an intrinsic ambiguity in the observed phenomena (72), which might adversely affect the intended performance (69).
ML models have the ability to determine implicitly any complex nonlinear relationship between independent and dependent variables (69), and have the ability to determine all possible interactions between predictor variables (73).	Overreliance on the capabilities of automation can lead to the related phenomenon of radiologist deskilling (74).
	Algorithms may be unreliable due to several technical constraints: domain adaptation is currently limited, and more solutions are required to help algorithms extrapolate well to new centers. Ultimately models may require calibration or retraining.
	Robustness to unintended data, such as artifacts, is also a technical constraint that needs to be overcome. Finally, the presence of more than one pathology (e.g., stroke or abscess associated with a tumor following treatment) can also confound algorithms as these cases are scarce and often unlabeled.
	Accuracy-driven performance metrics have led to a trend towards increasingly opaque models (73), although recent developments in interpretability and explainability may help to mitigate this to some extent (75).

Nonetheless, overall there appears to be good diagnostic performance of ML models using MRI features to differentiate between progressive disease and mimics. For now, if ML models are to be used they may be best confined to the centers where the data was obtained from, badged as research tools and undergo further improvement.

Concordant with a previous review of studies published up to Sept 2018 (11), the diagnostic performance of ML using implicit features did not appear to be superior to ML using explicit features. However, the small number of studies precluded meaningful quantitative comparison.

### 4.4 Implications for Clinical Practice and Future Research

The results demonstrate that glioblastoma treatment response monitoring biomarkers using ML are promising but are still at the early development stage and are not yet ready to be integrated into clinical practice. All studies would benefit from the improvements in methodology described above. Methodological profiles or standards might be developed through consortiums such as the European Cooperation in Science and Technology (COST) Glioma MR Imaging 2.0 (GliMR) (67) initiative or the ReSPOND Consortium (76). Determining an accurate reference standard for treatment response is challenging and performing prospective studies capturing contemporaneous detailed information on steroids and second line treatments is likely to mitigate the effects of confounding. Additionally, multiple image-localized biopsies at recurrence may lessen sampling bias due to PTRE and tumor admixture.

In future studies, it would be beneficial to perform analytical validation using external hold-out tests as epitomized by several studies in the current review. Using larger datasets which include a wider range of tumors and mimics as well as parameters from different sequences, manufacturers and coils, and thereby reduce overfitting, would also improve future studies. Multidisciplinary efforts and multicenter collaborations are therefore necessary (61). However, datasets will always be relatively small in neuro-oncological imaging even if distributed machine learning approaches such as federated learning, where the model comes to the data rather than the data comes to the model, overcome data sharing regulatory bottlenecks (61). Therefore, strategies to improve ML performance using small datasets, some of which are at the research stage, should be exploited further. Strategies include data augmentation (generate more varied image examples, within a single classification task) and the related process of meta-augmentation (generate more varied tasks, for a single example) (77) as well as transfer learning and the overlapping process of one- or few-shot learning (78). Transfer learning aims to learn representations from one domain (does not need to consist of brain tumors) and transfer the learned features to a closely related target domain (glioblastoma). Few-shot learning allows classifiers to be built from very small labelled training sets. Another research direction could be reducing the demand for image labelling. This field is known as self-supervised learning (79). Finally, an entirely different approach to counter the challenges of small datasets is to use synthetic data, for example using generative adversarial networks (80).

Predictions can also be made more informative through the modelling of prediction uncertainty including the generation of algorithms that would “know when they don’t know” what to predict (11).

Further downstream challenges for clinical adoption will be the completion of clinical validation (2) as well as the deployment of the clinical decision support (CDS) software to clinical settings. Clinical validation consists of evaluating the CDS software containing the locked machine learning model in a clinical trial thereby producing high level evidence (12). The CDS software deployment brings both technical and non-technical challenges. In terms of technical challenges, the CDS software must be easily integrated into the radiologist’s workflow (electronic health record system and picture archiving and communication system) and preferably deliver a fully automated process that analyzes images in real time and provides a quantitative and probabilistic report. Currently there has been little translation of CDS software into radiological departments however there are open source deployment solutions (71, 81).

Non-technical challenges relate to patient data safety and privacy issues; ethical, legal and financial barriers to developing and distributing tools that may impact a patient’s treatment course; medical device regulation; usability evaluation; clinical acceptance and medical education around the implementation of CDS software (14, 82). Medical education includes articulating the CDS software limitations to ensure there is judicious patient and imaging selection reflecting the cohort used for validation of the model (11).

## 5 Conclusion

A range of ML-based solutions primed as glioblastoma treatment response monitoring biomarkers may soon be ready for clinical adoption. To ensure clinical adoption, it would be beneficial during the development and validation of ML models that studies include large, well-annotated datasets where there has been meticulous consideration of the potential for confounding.

## Data Availability Statement

The original contributions presented in the study are included in the article/**Supplementary Material**. Further inquiries can be directed to the corresponding author.

## Author Contributions

TB: experimental design and implementation, analysis and interpretation of the data, performance accuracy statistical analysis, writing of draft manuscript, approval of the final version. AC, AR, AAB, CD, HS, AL, AM, BA, NM, JL, FV, KA, SO, MM: the implementation, analysis, and interpretation of the data and approval of the final version. MG: implementation, analysis and interpretation of the data, meta-analysis statistical analysis, writing of draft manuscript, approval of the final version. All authors contributed to the article and approved the submitted version.

## Funding

This research was supported by the Wellcome/EPSRC Centre for Medical Engineering (WT 203148/Z/16/Z) (TB, MG, AC, MM, SO) which includes open access fees, The Royal College of Radiologists (TB) and King’s College Hospital Research and Innovation (TB).

## Conflict of Interest

The authors declare that the research was conducted in the absence of any commercial or financial relationships that could be construed as a potential conflict of interest.

## Publisher’s Note

All claims expressed in this article are solely those of the authors and do not necessarily represent those of their affiliated organizations, or those of the publisher, the editors and the reviewers. Any product that may be evaluated in this article, or claim that may be made by its manufacturer, is not guaranteed or endorsed by the publisher.
