# Erratum: Imaging biomarkers of glioblastoma treatment response: a systematic review and meta-analysis of recent machine learning studies

**DOI:** 10.3389/fonc.2023.1217461

**Published:** 2023-05-24

**Authors:** Frontiers Production Office

**Affiliations:** Frontiers Media SA, Lausanne, Switzerland

**Keywords:** glioblastoma, machine learning, monitoring biomarkers, meta-analysis, artificial intelligence, treatment response, deep learning, glioma

Due to a production error, there was an error in the published **Table 1**. The 4th row of the table started at the second column instead of the first column, causing the contents of the last column to move to the next row, resulting in a formatting error. The corrected **Table 1** appears below.

**Table 1:** Studies using machine learning in the development of glioblastoma monitoring biomarkers.

Author	Target condition	Reference standard	Dataset(s)	Available demographic information	Methodology	Features selected	Test set performance
^a^Kim J.Y. et al. (34)	Early true progression or Early pseudoprogression	Mixture of histopathology and imaging follow up	Training = 61 Testing = 34 *T*_1_ C, FLAIR, DWI, DSC	Training = age mean ± SD (range) 58 ± 11 (34–83) male 38 (62%) Testing = age mean ± SD 62 ± 12 male 25 (74%) Data from Korea	Retrospective 2 centers: 1 train & 1 external test set. LASSO feature selection with 10-fold CV Linear generalized model	First-order, volume/shape, Second-order (texture), wavelet. ADC & CBV parameters included.	Recall 0.71 Specificity 0.90 Precision 0.83 BA 0.81 F1 0.77 AUC 0.85 (CI 0.71 – 0.99)
Kim J.Y. et al. (35)	Early true progression or Early pseudoprogression	Mixture of histopathology and imaging follow up	Training = 59 Testing = 24 *T*_1_ C, FLAIR, DTI, DSC	Training = age mean ± SD 61 ± 11 male 37 (63%) Testing = age mean ± SD 59 ± 12 male 9 (38%) Data from Korea	Retrospective 1 center LASSO feature selection with 10-fold CV Linear generalized model	First-order, Second-order (texture), wavelet. FA & CBV parameters included.	Recall 0.80 Specificity 0.63 Precision 0.36 BA 0.72 F1 0.50 AUC 0.67 (0.40 – 0.94)
Bacchi S. et al. (36)	True progression or PTRE (HGG)	Histopathology for progression and imaging follow up for pseudoprogression	Training = 44 Testing = 11 *T*_1_ C, FLAIR, DWI	Combined = age mean ± SD 56 ± 10 male 26 (47%) Data from Australia	Retrospective 1 center 3D CNN & 5-fold CV	CNN. FLAIR & DWI parameters	Recall 1.00 Specificity 0.60 Precision 0.75 BA 0.80 F1 0.86 AUC 0.80
Elshafeey N. et al. (37)	True progression or ^b^PTRE	Histopathology	Training = 98 Testing = 7 DSC, DCE	Training = age mean ± SD 50 ± 13 male 14 (58%) No testing demographic information Data from USA	Retrospective 3 centers mRMR feature selection. 1 test. 1) decision tree algorithm C5.0 2) SVM including LOO and 10-fold CV	K_trans_ & CBV parameters	Insufficient published data to determine diagnostic performance (CV training results available recall 0.91; specificity 0.88)
Verma G. et al. (38)	True progression or Pseudoprogression	Mixture of histopathology and imaging follow up	Training = 27 3D-EPSI	Training = age mean ± SD 64 ± 10 male 14 (52%) Data from USA	Retrospective 1 center Multivariate logistic regression LOOCV	Cho/NAA & Cho/Cr	No test set (CV training results available recall 0.94; specificity 0.87)
Ismail M. et al. (39)	True progression or Pseudoprogression	Mixture of histopathology and imaging follow up	Training = 59 Testing = 46 *T*_1_ C, *T*_2_/ FLAIR	Training = age mean(range) 61 (26–74) male 39 (66%) Testing = age mean (range) 56 (25–76) male 30 (65%) Data from USA	Retrospective 2 centers: 1 train & 1 external test set. SVM & 4-fold CV	Global & curvature shape	Recall 1.00 Specificity 0.67 Precision 0.88 BA 0.83 F1 0.94
^a^Bani-Sadr A. et al. (40)	True progression or Pseudoprogression	Mixture of histopathology and imaging follow up	Training = 52 Testing = 24 *T*_1_ C, FLAIR MGMT promoter status	Combined = age mean ± SD 58 ± 11 male 45 (59%) Data from France	Retrospective 1 center Random Forest.	Second-order features +/- MGMT promoter status	Recall 0.94 (0.71 - 1.00) Specificity 0.38 (0.09 - 0.76) Precision 0.36 BA 0.66 F1 0.84 AUC 0.77 & non-MRI: Recall 0.80 (0.56 - 0.94) Specificity 0.75 (0.19 - 0.99) Precision 0.86 BA 0.74 F1 0.83 AUC 0.85
Gao X.Y. et al. (41)	True progression or PTRE (HGG)	Mixture of histopathology and imaging follow up	Training = 34 Testing = 15 (per lesion) *T*_1_ C, FLAIR	Combined = age mean ± SD 51 ± 11 male 14 (36%) (per patient) Data from China	Retrospective 2 centers SVM & 5-fold CV	*T*_1_ C, FLAIR subtraction map parameters	Recall 1.00 Specificity 0.90 Precision 0.83 BA 0.95 F1 0.91 AUC 0.94 (0.78 – 1.00)
Jang B-S. et al. (42)	True progression or Pseudoprogression	Mixture of histopathology and imaging follow up	Training = 59 Testing = 19 *T*_1_ C & clinical features & IDH/MGMT promoter status	Training = age median (range) 56 (22–77) male 41 (70%) Testing = age mean ± SD 53 (28–75) male 10 (53%) Data from Korea	Retrospective 2 centers 1 train & 1 external test set. CNN LSTM & 10-fold CV (compared to Random Forest)	CNN *T*_1_ C parameters +/- Age; Gender; MGMT status; IDH mutation; radiotherapy dose and fractions; follow-up interval	Recall 0.64 Specificity 0.50 Precision 0.64 BA 0.57 F1 0.63 AUC 0.69 & non-MRI: Recall 0.72 Specificity 0.75 Precision 0.80 BA 0.74 F1 0.76 AUC 0.83
Li M. et al. (43)	True progression or ^b^PTRE	Imaging follow up	Training = 84 DTI	No demographic information Data from USA	Retrospective. 1 center DC-AL GAN CNN with SVM including 5 and 10 and 20-fold CV (compared to DCGAN, VGG, ResNet, and DenseNet)	CNN. DTI	No test set (CV training results only available: Recall 0.98 Specificity 0.88 AUC 0.95)
Akbari H. et al. (44)	True progression or Pseudoprogression	Histopathology	Training = 40 Testing = 23 Testing = 20 *T*_1_ C, *T*_2_/FLAIR, DTI, DSC, DCE	Combined internal = age mean (range) 57 (33–82) male 38 (60%) No external demographic information Data from USA	Retrospective 2 centers. 1 train & test. 1 external test set. imagenet_vgg_f CNN SVM & LOOCV	First-order, second-order (texture). CBV, PH, TR, *T*_1_ C, *T*_2_/FLAIR parameters included.	Recall 0.70 Specificity 0.80 Precision 0.78 BA 0.75 F1 0.74 AUC 0.80
Li X. et al. (45)	Early True progression or early pseudoprogression (HGG)	Mixture of histopathology and imaging follow up	Training = 362 *T*_1_ C, *T*_2_, multi-voxel & single-voxel 1H-MRS, ASL	Training = age mean (range) 50 (19–70) male 218 (60%) Data from China	Retrospective Gabor dictionary and sparse representation classifier (SRC)	Sparse representations	No test set (CV training results only available: Recall 0.97 Specificity 0.83)
Manning P et al. (46)	True progression or pseudoprogression	Mixture of histopathology and imaging follow up	Training = 32 DSC, ASL	Training = age mean ± SD 56 ± 13 male 22 (69%) Data from USA	Retrospective 1 center Linear discriminant analysis & LOOCV	CBF and CBV parameters included.	No test set (CV training results only available: Recall 0.92 Specificity 0.86 AUC 0.95)
Park J.E. et al., 2020 (47)	Early True progression or early pseudoprogression	Mixture of histopathology and imaging follow up	Training = 53 Testing = 33 *T*_1_ C	Training = age mean ± SD 56 ± 11 male 31 (59%) Testing = age mean ± SD 62 ± 12 male 25 (76%) Data from Korea	Retrospective 2 centers. 1 train & test. 1 external test set. Random Forest feature selection with 10-fold CV (Automated segmentation)	First-order, volume/shape, Second-order (texture), wavelet parameters included.	Recall 0.61 Specificity 0.47 Precision 0.58 BA 0.54 F1 0.59 AUC 0.65 (0.46 – 0.84)
Lee J. et al. (48)	True progression or ^b^PTRE (HGG)	Histopathology	Training = 43 *T*_1,_ *T*_1_ C, *T*_2,_ FLAIR, (subtractions: *T*_1_ C - *T*_1_, *T*_2-_ FLAIR) ADC parameters.	Training =age mean ± SD (range) 52 ± 13 (16–74) male 24 (56%) Data from USA	Retrospective 1 center CNN-LSTM. 3-fold CV	CNN-LSTM parameters.	No test set (CV training results only available: AUC 0.81 (0.72 - 0.88))
Kebir S. et al. (49)	True progression or ^b^PTRE	Imaging follow up	Training = 30 Testing = 14 O-(2[^18^F]-fluoroethyl)-L-tyrosine (FET)	Combined = age mean ± SD (range) 57 ± 11 (34-79) male 34 (77%) Data from Germany	Retrospective 1 center Linear discriminant analysis. 3-fold CV	TBR_mean_ TBR_max_ TTP_min_ parameters.	Recall 1.00 Specificity 0.80 Precision 0.90 BA 0.92 F1 0.95 AUC 0.93 (0.78 - 1.00)
Cluceru J. et al. (50)	Early True progression or early pseudoprogression (HGG)	Histopathology	Training = 139 DSC, MRSI, DWI, DTI	Training = age median (range) 52 (21–84) Male 83 (60%) Data from USA Ethnicity: White 112 (80%) American Indian 1 (1%) Asian 6 (4%( Pacific Islander 2 (1%) Other 18 (13%)	Retrospective 1 center Multivariate logistic regression. 5-fold CV	Cho, Cho/Cr, Cho/NAA & CBV parameters.	No test set (CV training results only available: Recall 0.65 (0.33 - 0.96); Specificity 0.62 (0.21 - 1.00) AUC 0.69 (0.51 - 0.87))
Jang B.S. et al. (51)	True progression or ^b^PTRE	Mixture of histopathology and imaging follow up (including PET)	(i) (trained model = 78) testing = 104 (ii) all training = 182 *T*_1_ C & clinical, molecular, timings, radiotherapy data	Testing = age median (range) 55 (25-76) male 59 (67%) Data from Korea	Retrospective (i) 6 centers 1 external test set. CNN LSTM (ii) 7 centers 1 training set CNN LSTM & 10-fold CV	CNN *T*_1_ C parameters and Age; Gender; MGMT status; IDH mutation; radiotherapy dose and fractions; follow-up interval	(i) Insufficient published data to determine diagnostic performance (ii) No test set (CV training results available AUPRC 0.87)

a Within publication some data appears mathematically discrepant.

b Within publication discrepant or unclear information (e.g. interval after radiotherapy).

Unless otherwise stated, glioblastoma alone was analyzed.

PTRE, post-treatment related effects; HGG, high-grade glioma.

MRI sequences: T_1_ C, postcontrast T_1_-weighted; T_2_, T_2_-weighted; FLAIR, fluid-attenuated inversion recovery; DSC, dynamic susceptibility-weighted; DCE, dynamic contrast-enhanced; DWI, diffusion-weighted imaging; DTI, diffusor tensor imaging; ASL, arterial spin labelling; MRI parameters: ADC, apparent diffusion coefficient; FA, fractional anisotropy; TR, trace (DTI); CBV, cerebral blood volume; PH, peak height; K_trans_, volume transfer constant.

Magnetic resonance spectroscopy: 1H-MRS, 1H-magnetic resonance spectroscopy; 3D-EPSI, 3D echo planar spectroscopic imaging.

1H-MRS parameters: Cr, creatine; Cho, choline; NAA, N-acetyl aspartate.

Nuclear medicine: TBR, tumor-to-brain ratio; TTP, time-to-peak.

Molecular markers: MGMT, O6-methylguanine-DNA methyltransferase; IDH, isocitrate dehydrogenase.

Machine learning methodology: CV, cross validation; LOOCV, leave-one-out cross validation; SVM, support vector machine; CNN, convolutional neural network; LASSO, least absolute shrinkage and selection operator; LSTM, long short-term memory; mRMR, minimum redundancy and maximum relevance; VGG, Visual Geometry Group (algorithm); DCGAN, deep convolutional generative adversarial network; DC-AL GAN, DCGAN with AlexNet.

Statistical measures: CI, confidence intervals; BA, balanced accuracy; AUC, area under the receiver operator characteristic curve; AUPRC, area under the precision-recall curve.

The publisher apologizes for this error. The original version of this article has been updated.

