# Supplementary material for: Imaging Biomarkers of Glioblastoma Treatment Response: A Systematic Review and Meta-Analysis of Recent Machine Learning Studies
Source: Front Oncol. 2022 Jan 31;12:799662. doi: 10.3389/fonc.2022.799662 (PMC8842649; doi:10.3389/fonc.2022.799662)
Supplement: Supplementary file 1 [file DataSheet_1.docx]

Supplementary Material

**Supplementary Table S1.** MEDLINE, EMBASE and Cochrane Register search strategies.

| MEDLINE (OVID). PubMed was included.  The search strategy for Title/Abstract terms used a combination of subject headings (MeSH terms) and keywords:  Database: Ovid MEDLINE(R)  Search Strategy:  1 exp Glioblastoma/  2 high grade glioma.mp.  3 pseudoprogression.mp.  4 imaging.mp.  5 exp Magnetic Resonance Imaging/ or MRI.mp.  6 pet.mp.  7 exp Positron-Emission Tomography/  8 1 or 2 or 3  9 4 or 5 or 6 or 7  10 8 and 9  Strategies requiring AND “radiomics OR artificial intelligence OR machine learning OR neural networks OR computer OR deep learning OR monitoring biomarker OR treatment response” were insensitive and not used in final search. |
| --- |
| EMBASE (OVID).  Subject headings and keywords:  Database: Embase  Search Strategy:  1 exp Glioblastoma/  2 high grade glioma.mp.  3 pseudoprogression.mp.  4 exp multiparametric magnetic resonance imaging/ or exp imaging/ or exp nuclear magnetic resonance imaging/  5 magnetic resonance imaging.mp.  6 MRI.mp.  7 PET.mp. or exp positron emission tomography/  8 1 or 2 or 3  9 4 or 5 or 6 or 7  10 8 and 9  11 limit 10 to exclude medline journals  Insensitive strategies were not used in final search. |
| Cochrane Register.  Epistemonikos review database included, protocols included, CENTRAL (Cochrane central register of controlled trials included which includes <https://www.ebscohost.com/nursing/products/cinahl-databases>, <https://clinicaltrials.gov>, <https://www.who.int/ictrp/en/>).  Subject headings and keywords:  #1 MeSH descriptor: [Glioblastoma] explode all trees  #2 high grade glioma  #3 pseudoprogression  #4 imaging  #5 MeSH descriptor: [Magnetic Resonance Imaging] explode all trees  #6 MRI  #7 PET  #8 MeSH descriptor: [Positron-Emission Tomography] explode all trees  #9 {OR #1-#3}  #10 {OR #4-#8}  **#11 {AND #9-#10}** |
| Health Technology Assessment. <https://database.inahta.org/>  Subject headings and keywords:  (("Glioblastoma"[mh]) OR (high grade glioma) OR (pseudoprogression))  No results. |

**Supplementary Statistical Information.**

*Challenges related to meta-analysis of diagnostic studies*

Meta-analyses of the data from diagnostic accuracy studies require a statistical approach in which the interdependence of primary diagnostic measures is included and adjusted for as the values of key diagnostic measures (sensitivity and specificity) are usually highly related one to another through the cut-off value.^1-3^ Meta-analyses of the data from diagnostic accuracy studies also require adjustment for a relatively high level of heterogeneity commonly observed among the results of such studies. The heterogeneity is likely to be the effect of at least three factors which are: (1) differences in applied cut-off values, (2) differences in the examined groups of patients, and (3) the random error. Since all these factors are likely non-negligible the statistical model deriving summary estimates of diagnostic accuracies should also take them into account.

In order to address the issues mentioned above several statistical models have been proposed.^3-7^ Among them two approaches offer statistically rigorous solutions that overcome known limitations of earlier methods^2^ and turned out to be implementable in popular statistical packages such as STATA, SAS or R. These are the 2001 hierarchical regression approach (HSROC model)^6^ and the 2005 bivariate model^7^ which have been used widely and considered the reference standard for meta-analyses of diagnostic measures. Although these two approaches model diagnostic accuracies in different ways they become equivalent when no covariates are included in the model.^8^ In this meta-analysis the bivariate model was selected.


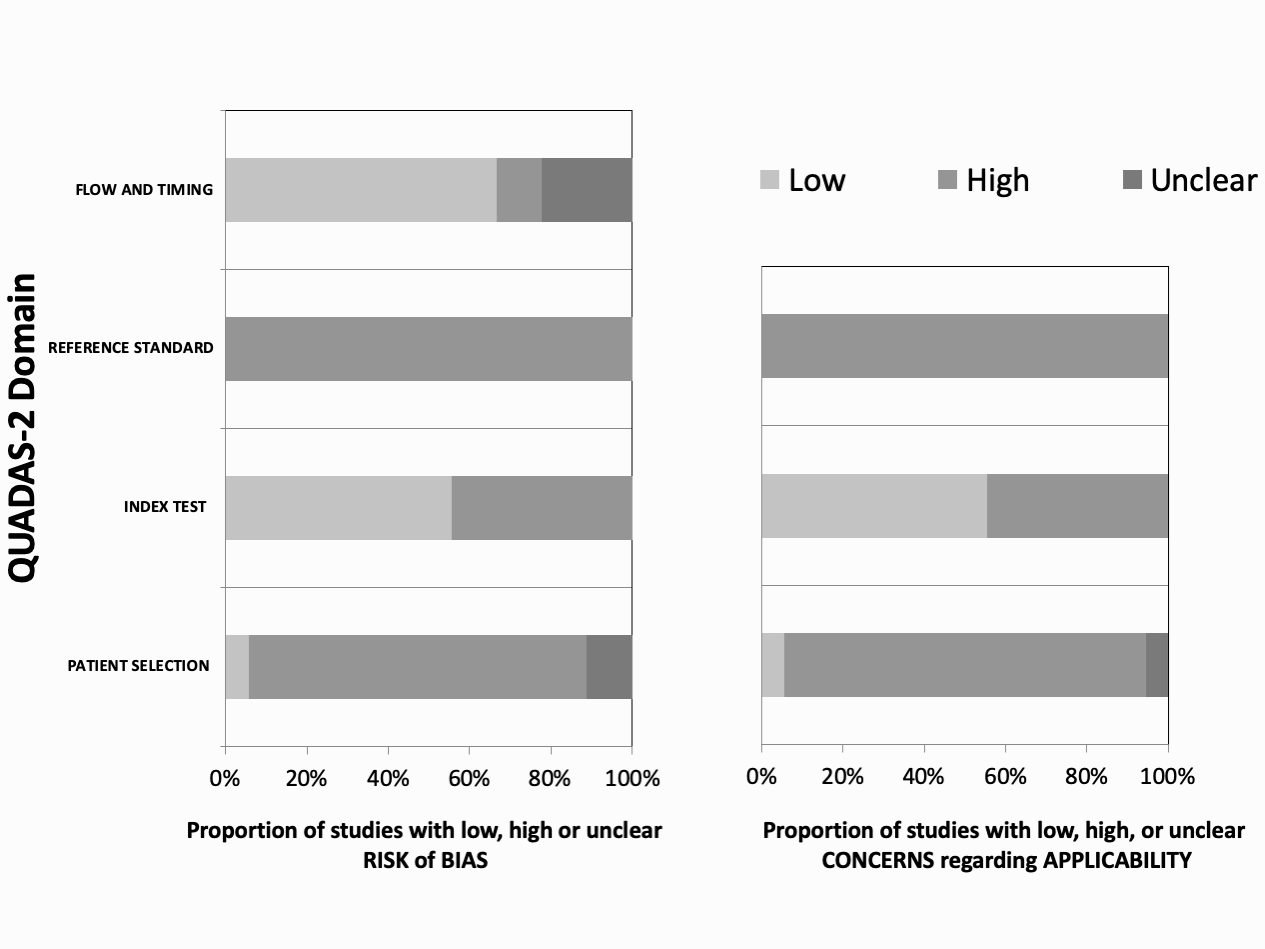


**Supplementary Figure S1.** Bar chart showing risk of bias and concerns of applicability assessment for the 18 monitoring biomarker studies included in the bias assessment.


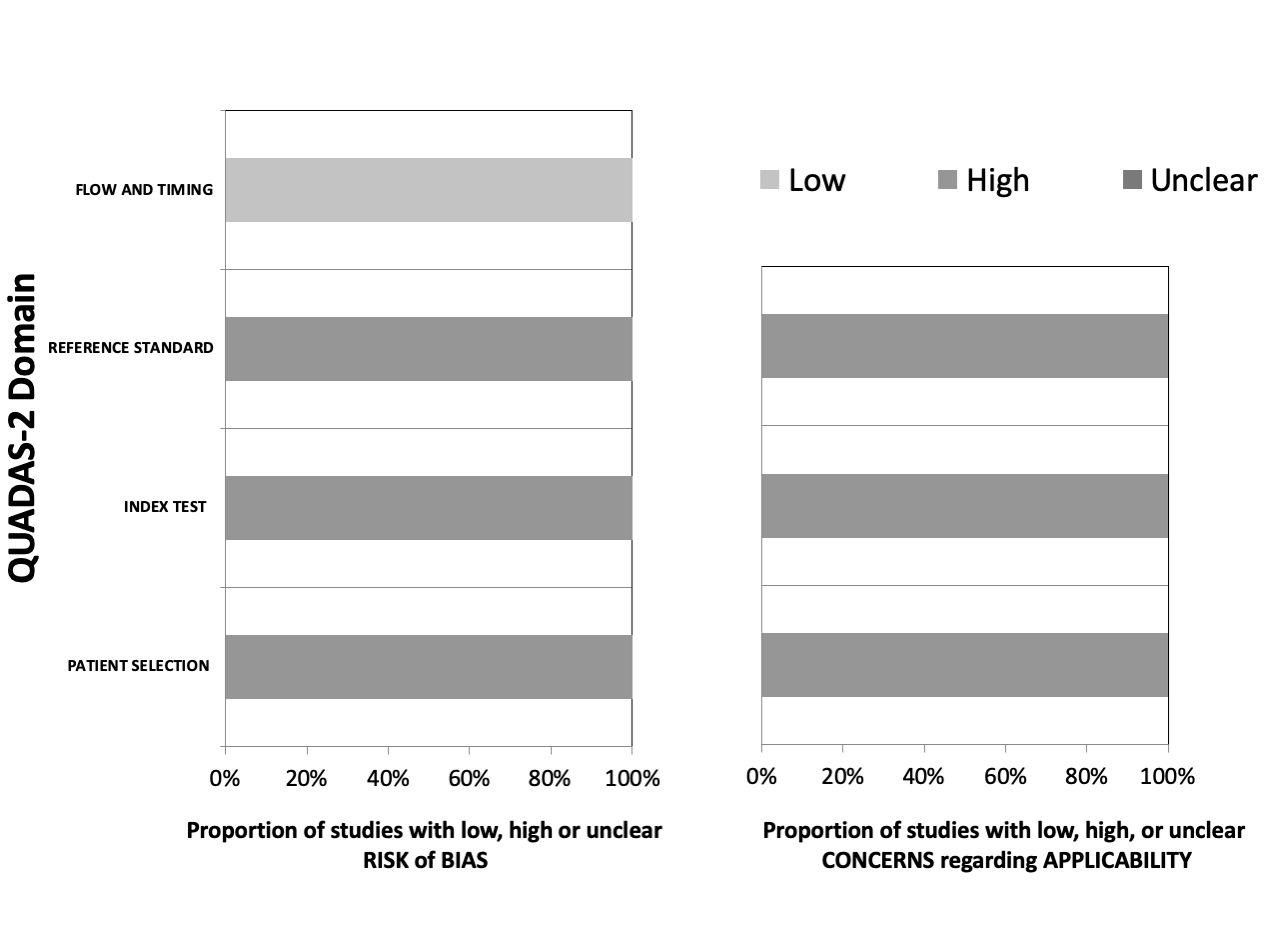


**Supplementary Figure S2.** Bar chart showing risk of bias and concerns of applicability assessment for the 3 prognostic biomarker studies included in the bias assessment.

**References**

1. Zhou X-H, Obuchowski NA, McClish DK. Statistical methods in diagnostic medicine. Second Edition. Chicester: John Wiley & Sons (2011) 55 p.

2. Harbord RM, Whiting P. *Metandi: Meta-analysis of Diagnostic Accuracy Using Hierarchical Logistic Regression.*The Stata Journal. (2009) 9(2):211-229.

3. Schwarzer G, Carpenter JR, Rücker,G. *Meta-Analysis with R*. Heidelberg: Springer (2015). 252 p.

4. Willis B, Quigley M. *Uptake of newer methodological developments and the deployment of meta-analysis in diagnostic test research: a systematic review.* BMC Med Res Methodol. (2011) 11:27

5. Moses LE, Shapiro D, Littenberg B. *Combining independent studies of a diagnostic test into a summary ROC curve: Data-analytic approaches and some additional considerations.* Stat Med. (1993) 12(14):1293–1316.

6. Rutter CM, Gatsonis CA. *A hierarchical regression approach to metaanalysis of diagnostic test accuracy evaluations.* Stat Med. (2001) 20:2865–2884.

7. Reitsma J B, Glas AS, Rutjes AWS, Scholten RJPM, Bossuyt PM, Zwinderman AH. *Bivariate analysis of sensitivity and specificity produces informative summary measures in diagnostic reviews.* J Clin Epidemiol. (2005) 58:982–990.

8. Arends L, Hamza T, Van Houwelingen J, Heijenbrok-Kal M, Hunink M, Stijnen T. *Bivariate Random Effects Meta-Analysis of ROC Curves.* Med Decis Making (2008) 28:621-638.
